# A Scoping Review of Pediatric Leukemia and Oral Health: Contemporary Approaches and Oncodental Insights

**DOI:** 10.7759/cureus.97707

**Published:** 2025-11-24

**Authors:** Nupur Ninawe, Snehal Jagtap, Ritesh Kalaskar, Shruti Balasubramanian, Surendra Bahetwar, Naveen Reddy Banda

**Affiliations:** 1 Pediatric Dentistry, Government Dental College and Hospital, Nagpur, Nagpur, IND; 2 Pediatric Dentistry, ITS Dental College, Greater Noida, IND; 3 Pediatric Dentistry, Ibn Sina National College, Jeddah, SAU

**Keywords:** dental management, dental management in childhood cancer, leukemia, oncodentistry, oral care, pediatric leukemia

## Abstract

One of the most common cancers in children is pediatric leukemia, especially acute lymphoblastic leukemia. Survival rates have grown dramatically because of advancements in combination chemotherapy; nonetheless, treatment-related problems, particularly those affecting the oral cavity, pose substantial hurdles. Oral symptoms, including mucositis, xerostomia, and ulcerations, not only cause discomfort and interfere with food intake but can also result in systemic infections that interrupt the course of cancer therapy. The purpose of this review is to synthesize existing knowledge regarding the prevalence, clinical evaluation, and management of oral tissue complications in pediatric leukemia patients. It emphasizes the importance of prevention, early intervention, and interprofessional collaboration, while also exploring how oral health can be integrated into pediatric oncology treatment. To achieve this, a comprehensive search of peer-reviewed literature was carried out across major electronic databases to identify studies related to oral health issues in pediatric leukemia. Articles were reviewed and selected according to inclusion criteria that focused on clinical symptoms, preventive strategies, therapeutic approaches, and multidisciplinary care models. The findings revealed notable discrepancies in the implementation of standardized oral care practices. Research indicates that preventive dental plans and early management are effective in reducing oral complications and improving adherence to cancer treatment; however, there remain deficiencies in the integration of standardized protocols and in the training provided to caregivers.

## Introduction and background

Pediatric leukemia, particularly acute lymphoblastic leukemia (ALL), is the most common childhood cancer, accounting for over 25% of all pediatric malignancies [1]. Advances in combination chemotherapy have significantly improved survival rates in pediatric leukemia. However, treatment-related complications, particularly those involving the oral cavity, continue to adversely affect the comfort and overall quality of life of young patients [2].

Oral side effects, such as mucositis, xerostomia, and ulcerations, are frequent and can cause pain, eating difficulties, and nutritional issues. More critically, they can also lead to systemic infections, posing a risk to the continuity and success of cancer therapy [1,2]. Recognizing this, oncodentistry has emerged as a vital part of pediatric oncology. Yet, the integration of standardized oral care protocols throughout the cancer treatment process remains inconsistent.

This highlights the need for interdisciplinary collaboration, supports evidence-based preventive strategies, and emphasizes child-friendly educational tools that empower both patients and caregivers. By addressing existing gaps in practice, this review advocates for a holistic, patient-centered approach to oral health in pediatric oncology, one that aims to minimize complications, support treatment continuity, and enhance the overall well-being of children undergoing leukemia treatment [3,4].

Methodology

Search Strategy

A comprehensive literature search was conducted to identify peer-reviewed publications relevant to the oral management of pediatric patients diagnosed with leukemia, with a specific emphasis on ALL, a major type in children. The search included multiple electronic databases, including PubMed and Google Scholar, using a combination of Medical Subject Headings (MeSH) and keywords such as: “Leukemia,” “Precursor Cell Lymphoblastic Leukemia-Lymphoma,” “Child,” “Pediatric Dentistry,” “Mucositis,” “Stomatitis,” “Mouth Diseases/Prevention and Control,” “Oral Health,” and “Antineoplastic Agents/Adverse Effects.”

The eligibility of studies was determined based on well-defined inclusion and exclusion criteria, which are presented in Table 1.

**Table 1:** Inclusion and exclusion criteria for article selection.

Inclusion criteria	Exclusion criteria
Studies involving children or adolescents diagnosed with leukemia, particularly acute lymphoblastic leukemia (ALL)	Studies involving adult patients with leukemia
Articles that specifically addressed the management of oral manifestations resulting from chemotherapy or cancer treatment	Articles not focusing on oral health or oral manifestations
Publications in English, published in peer-reviewed journals	Non-English publications or unpublished theses/dissertations
Systematic reviews, narrative reviews, clinical guidelines, or original research focused on dental care across different phases of leukemia treatment (pre-treatment, during therapy, and post-treatment)	Case reports, conference abstracts, commentaries, or editorials

Data Synthesis

To establish a structured, evidence-based framework for the dental management of pediatric leukemia patients, the selected literature was critically reviewed and synthesized. Extracted data were organized into thematic domains: (1) preventive oral strategies, (2) chemotherapy-related manifestations, and (3) multidisciplinary management models. Studies were qualitatively synthesized, and recurrent outcomes were tabulated to identify consensus and evidence gaps. This approach allowed comparison of heterogeneous data without performing a meta-analysis.

## Review

Background

Leukemia in children, especially ALL, is characterized by chromosomal abnormalities and disrupted development of lymphoid precursor cells. The highest incidence is seen in children aged one to four years [1]. While advancements in systemic chemotherapy have significantly improved survival rates, mucotoxic side effects remain a major clinical challenge in pediatric care [1].

Oral symptoms, such as gingival swelling, spontaneous bleeding, ulcerations, and tooth mobility, often appear before a hematologic diagnosis is confirmed [2]. Chemotherapeutic agents like methotrexate and doxorubicin can compromise oral mucosal integrity, reduce immune defense, and disrupt the oral microbiome, increasing the risk of opportunistic infections, mucositis, and systemic sepsis [5]. These complications not only impact nutrition and psychological well-being but can also lead to treatment delays and poor adherence.

To address this, oncodentistry has emerged as a specialized field integrating preventive and supportive oral care during cancer therapy [3]. However, many studies reveal that oral care is still inconsistently included in pediatric oncology protocols. The Multinational Association of Supportive Care in Cancer (MASCC) guidelines recommend routine oral hygiene education and preventive strategies, but traditional approaches often fall short, especially in children with cognitive or emotional barriers [6,7].

To better monitor and manage these complications, several validated assessment tools have been developed, including the WHO functional scale, National Cancer Institute's (NCI) ability-based scale, and pediatric-specific tools like the Children’s International Mucositis Evaluation Scale (ChIMES), Oral Assessment Guide (OAG), and its modified versions [8,9]. Additional tools like the Oral Mucositis Evaluation Scale (OMAS), the Oral Mucosal Daily Questionnaire, and Children’s Oncology Group guidelines provide structured approaches for intervention [10].

Despite this framework, oral care remains fragmented across therapy phases, i.e., induction, consolidation, maintenance, and remission. Limited involvement of pediatric dentists and poor interprofessional coordination increase the risk of severe outcomes, including central line-associated bloodstream infections (CLABSIs) originating from untreated oral infections [4].

The purpose of this study is to critically analyze and consolidate up-to-date data on the prevalence, severity, and management of oral complications in children with leukemia. It emphasizes the urgent need for proactive preventive strategies, innovative behavior management techniques, and seamless multidisciplinary collaboration to ensure effective, continuous oral care throughout the cancer treatment journey.

Among the pediatric cancers, leukemia stands out as both the most frequent and the most disruptive to a child's health, with ALL and acute myeloid leukemia (AML) accounting for the majority of cases. While the diagnosis is devastating for families, it often explains the subtle and sometimes dramatic changes that have appeared in the child’s mouth. Many children with undiagnosed leukemia first display oral symptoms, such as swollen gums, spontaneous bleeding, and persistent ulcerations, long before laboratory results confirm their illness [2].

Once therapy begins, the interplay between disease and treatment becomes even more complex. ALL, the predominant form, is treated with intensive, multi-agent chemotherapy regimens. Interestingly, children who receive careful dental monitoring sometimes fare better in terms of oral hygiene, developing fewer carious lesions than their peers, possibly due to the vigilance and structured care imposed by cancer protocols [11]. But this apparent benefit is offset by the harsh realities of chemotherapy. Cytotoxic agents, needed to suppress malignant cells, unfortunately, do not spare the healthy oral tissues. The delicate lining of the mouth is especially vulnerable: mucositis, xerostomia, and a drop in salivary pH emerge as predictable consequences of the treatment, each compounding the risk of infection, ulceration, and oral pain.

For pediatric patients, the timing of chemotherapy is more than a technical detail. When these medications are administered during periods of dental development, disturbances are almost inevitable; thin or pitted enamel, shortened roots, delayed eruption, and irregularly positioned teeth can follow. Such changes rarely resolve on their own, lingering as functional and cosmetic concerns long after cancer has been conquered [12].

If ALL is the most common, AML is arguably the most dramatic in its oral presentations. Children with AML can show gingival hypertrophy, spontaneous bleeding, and petechiae before any oncologist has been consulted, sometimes prompting the dentist to become the first to suspect leukemia [13]. Because AML more aggressively suppresses normal blood cell production, the tissues of the mouth become sites of infection, and excessive bleeding occurs, often before anti-leukemic therapy even begins. As treatment proceeds, the pervasive immunosuppression, coupled with compromised mucosa, opens the door for severe periodontal disease, enamel discoloration, and other opportunistic infections. While research continues to clarify whether these children have higher rates of periodontal disease than their healthy peers, it is undisputed that poor oral hygiene and a weakened immune system, together, magnify the risk of both caries and gum disease in either form of leukemia [14].

Thus, the journey through pediatric leukemia is not just about the disease in the bloodstream but also about the story told inside the mouth. The double assault, first from the cancer and then from the treatments meant to cure it, renders children extraordinarily vulnerable to oral complications. In this context, the role of tailored dental care and a close alliance between oncologists and oral health professionals becomes clear. Only through this collaboration can we hope to reduce complications and help children regain both their smiles and their health.

Oral manifestations as clinical indicators in pediatric leukemia

Oral signs can serve as early indicators of pediatric leukemia, sometimes even preceding hematological abnormalities and providing diagnostic and prognostic clues during disease onset or relapse. In AML, oral symptoms may appear before diagnosis in many cases, with up to 90% of pediatric AML patients exhibiting manifestations such as gingival enlargement, petechiae, mucosal pallor, spontaneous bleeding, ulcerations, hemorrhagic bullae, and necrosis, primarily caused by leukemic infiltration and thrombocytopenia [13,15]. In contrast, ALL often presents with oral complications secondary to chemotherapy toxicity and immune suppression, including herpetic lesions, candidiasis, angular cheilitis, mucositis (which notably impacts eating, comfort, and oral hygiene), and xerostomia. The severity of mucositis is commonly assessed using standardized tools such as the World Health Organization (WHO) Oral Toxicity Scale and ChIMES.

Soares et al. (2023) [16] highlighted that oral lesions can act as early red flags in pediatric ALL, emphasizing the importance of frequent oral examinations since neutropenia and mucosal barrier breakdown predispose patients to rapidly spreading oral infections that may escalate into systemic complications. Xerostomia and altered salivary pH during chemotherapy contribute to dental caries, halitosis, and microbial overgrowth, with children affected by ALL often showing reduced salivary flow and increased mucosal sensitivity compared to healthy peers [11].

In summary, oral manifestations are not just local effects; they often reflect systemic disease activity or treatment-related toxicity. Early identification and management of these symptoms are crucial for improving clinical outcomes, reducing complications, and enhancing the overall prognosis in pediatric leukemia care.

Leukemia, particularly ALL, and its treatment protocols have profound implications for oral health in pediatric patients. Chemotherapy regimens, such as the Acute Lymphoblastic Leukemia Intercontinental Berlin-Frankfurt-Münster (ALL IC-BFM 2009) protocol, can significantly alter the oral environment. Commonly observed complications include oral mucositis, xerostomia, gingivitis, candidiasis, stomatitis, and dental caries. These arise due to cytotoxic effects on oral tissues, decreased salivary flow, disrupted microbial flora, and immune suppression, all of which reduce a child’s capacity to maintain effective oral hygiene.

For instance, Shayani et al. (2022) found that children undergoing chemotherapy for ALL experienced a 69.57% incidence of gingivitis and an increased caries risk (RR = 2.15; 95% CI: 1.34-3.45) compared to children without leukemia or chemotherapy exposure [17]. These oral complications significantly impact nutrition, speech, and quality of life, and can lead to treatment delays or hospital readmission.

Additionally, the advent of novel targeted therapies in AML, such as venetoclax, midostaurin, enasidenib, and oral azacitidine, has introduced new oral toxicities. Side effects like mucosal sensitivity, stomatitis, and delayed healing have been noted [18,19]. These agents also pose drug interaction risks with commonly used dental medications such as antibiotics, antifungals, and analgesics, emphasizing the need for interdisciplinary collaboration between oncologists and dental teams.

Thus, recognizing and managing both conventional chemotherapy-induced and novel agent-associated oral complications is critical in pediatric leukemia care. Preventive strategies, early intervention, and personalized dental protocols must be integrated into oncology pathways to safeguard oral health and enhance patient outcomes.

Long-term dental and developmental sequelae may arise when anti-leukemic therapy is administered during critical stages of dental development, resulting in lasting effects such as root shortening, delayed eruption, enamel hypoplasia, hypodontia, microdontia, and craniofacial growth disturbances.

These issues can impair function, esthetics, and psychological well-being in survivors, often requiring comprehensive dental and orthodontic care. Thus, dental professionals play a vital role in bridging curative oncology with preventive and restorative dentistry, ensuring that children not only survive leukemia but also enjoy an improved quality of life.

Structured dental maintenance during leukemia treatment

As pediatric leukemia treatments become more intensive, with protocols like hematopoietic stem cell transplantation (HSCT) and oral antineoplastics, structured dental care is increasingly essential. Oral complications such as mucositis, infections, and myeloid sarcoma (MS) can interfere with treatment, delay healing, or even signal relapse. Proactive dental protocols, tailored to each treatment phase, are crucial to reduce these risks.

Oral mucositis (OM) is one of the most frequent and painful side effects, especially during HSCT conditioning. Symptoms begin within three to four days after infusion, peak as painful ulcers by days seven to 14, and typically resolve within two to three weeks [20]. OM presents with erythema, swelling, mucosal atrophy, and ulcerations, severely impacting nutrition, speech, and oral hygiene. Risk factors include younger age, low BMI, female sex, and T-cell leukemia subtype [21]. Preventive strategies like professional dental care (PDC) and photobiomodulation (PBM) significantly reduce OM severity, improving patient comfort and adherence to therapy.

Oral MS, a rare tumor of immature myeloid cells, can present as gingival swelling, ulcers, or non-specific soft tissue lesions, often misdiagnosed as common dental infections like abscesses or pericoronitis [22]. Delays in recognizing primary oral MS can postpone leukemia diagnosis, as its average detection time is five times longer than that of secondary MS, underlining the importance of dental professionals in early detection and referral.

Oral side effects also impact treatment adherence. During ALL maintenance therapy, oral chemotherapy is often administered at home. However, parental difficulty in managing medication schedules can compromise adherence [23]. Dentists play a supportive role in monitoring oral symptoms and guiding families through treatment.

Additionally, chemotherapy-induced nausea and vomiting (CINV), even with antiemetic use, can reduce oral intake and complicate hygiene routines. Understanding acute vs. delayed emesis helps dental teams adjust care strategies to protect enamel, regulate pH, and support hydration [24].

Given the diverse nature of oral complications, a phased dental care approach is essential, which is as follows. Pre-treatment: Eliminate infection sources, conduct a full dental assessment, and educate caregivers. During treatment: Manage OM, reinforce hygiene, and coordinate with oncologists. Post-treatment: Address long-term effects like enamel defects, hypodontia, and root anomalies. This structured model ensures fewer treatment interruptions, improved clinical outcomes, and better quality of life (Table 2). Dental professionals are key members of the multidisciplinary pediatric oncology team.

**Table 2:** Structured dental care across leukemia treatment phases.

Treatment phase	Oral health goals	Common complications	Recommended dental interventions	Supporting references
Pre-treatment phase	Remove infectious sources, educate patients/caregivers, and create a baseline for oral health	Existing periodontitis/caries and gingival inflammation	Oral hygiene instruction, comprehensive dental examination, fluoride therapy, and extraction of non-restorable teeth	American Academy of Pediatric Dentistry (2024) [25]; Hong & da Fonseca (2008) [26]
Induction/intensive chemotherapy	Prevent/control mucositis, sustain hygiene during immunosuppression	Oral mucositis, xerostomia, fungal infection, and gingival bleeding	Professional dental care (saline/bicarbonate rinses, photobiomodulation as indicated) and antifungals as needed	Miranda-Silva et al. (2022) [21]; Holdsworth et al. (2006) [24]
Maintenance therapy	Monitor long-term toxicity, support adherence to oral chemotherapy	Demineralization, gingivitis, and adherence difficulty	Routine follow-ups, behavior reinforcement for home care, and coordinate with oncology for meds	Tang et al. (2022) [23]
Hematopoietic stem cell transplantation (HSCT)	Reduce oral mucositis & infection, preserve mucosal integrity	Significant oral mucositis, painful ulcers, and opportunistic infections	Photobiomodulation prophylaxis for oral mucositis, intensive hygiene schedule, soft and non-irritating diet	Eduardo et al. (2015) [20]; Miranda-Silva et al. (2022) [21]
Post-treatment/long-term follow-up	Manage developmental anomalies, restore function & esthetics, psychosocial support	Hypodontia/microdontia, enamel hypoplasia, and root shortening	Pedodontic care, restorative & orthodontic planning, ongoing fluoride	Miranda-Silva et al. (2022) [21]; Shayani et al. (2022) [17]

Cross-disciplinary collaboration: Oncodental team strategies for maintaining oral health in pediatric leukemia

Dental disease remains the most prevalent non-communicable condition globally and poses a significant risk to pediatric oncology patients, especially those receiving radiation therapy or bone-modifying agents. In such cases, compromised oral health can increase the likelihood of serious complications, including medication-related osteonecrosis of the jaw (MRONJ) and osteoradionecrosis (ORN). Implementation of preventive dental protocols has been shown to significantly reduce these risks.

The integration of routine dental care into pediatric oncology is critical for improving long-term health outcomes. Multidisciplinary collaboration ensures not only the successful management of oncologic disease but also the preservation of the child's oral function and quality of life. The experiences of institutions establishing dental oncology units demonstrate that multidisciplinary leadership and well-defined care pathways are essential for effective delivery of integrated services (Table 3) [25].

**Table 3:** Dental team's responsibilities in pediatric leukemia care.

Phase of cancer therapy	Objectives	Dental team's roles & actions	Supporting references
Before initiation of cancer therapy	Identify & eliminate sources of infection and avoid delays in cancer therapy	Comprehensive dental/oral exam, extract non-restorable/infected teeth, treat caries/periodontal disease, and communicate treatment plan with oncology	Hong & da Fonseca (2008) [26]
During immunosuppression (active therapy)	Maintain oral hygiene, manage side effects, and educate on hygiene importance	Monitor for mucositis, candidiasis, and bleeding, provide rinses, pain relief, guide gentle hygiene, and avoid invasive procedures	Byrne et al. (2024) [27] Bavier (1990) [28]
After completion of cancer therapy	Restore oral health and reinforce habits. Evaluate long-term complications (root stunting, xerostomia, microdontia)	Restorative & orthodontic care as needed, patient/parent education	Little et al. (2012) [29]
Continuous responsibilities	Prevent/manage complications, coordinate with the oncology team, and provide lifelong dental education	Customized oral care plan, communication with oncologists, and ongoing reinforcement of care practices	da Fonseca (1998) [30]

Despite socioeconomic challenges, such as relatively unchanged infant mortality rates (85 per 1000 live births in 1991 vs. 74 in 2000), systematic improvements have been observed in pediatric oncology care over the past 15 years [31]. International experts emphasize the importance of local needs assessment, community involvement, collaboration with established cancer centers, formation of multidisciplinary healthcare teams, enhanced supportive care, financial support for travel and lodging, and the development of tailored treatment protocols suited to regional capacities [32].

Personalized dental management protocols for pediatric leukemia patients undergoing chemotherapy

Oral care for pediatric cancer patients must be personalized based on age, stage of dental development, and the timing and intensity of chemotherapy (CMT). Effective oral hygiene, reinforced by tailored dental protocols, is essential to reduce the risk of infection, support nutritional health, and preserve long-term oral function and esthetics. Studies show that proactive dental planning and consistent oral hygiene practices can mitigate many of the long-term complications of CMT and significantly enhance quality of life [33].

The following structured clinical protocol outlines the key dental considerations and interventions throughout the cancer treatment journey.

Comprehensive Pre-treatment Completion

Ideally, all necessary dental procedures should be completed prior to the initiation of cancer therapy. Temporary restorations may be used when immediate completion is not feasible, and non-urgent procedures should be deferred until hematologic parameters stabilize.

Prioritization of Urgent Dental Needs

Immediate attention must be given to symptomatic caries, irreversible pulpitis, and lesions posing systemic risk. Treatment includes elimination of infections, extractions of non-restorable teeth, correction of defective restorations, and removal of mucosal irritants.

Endodontic Management in Primary Teeth

Pulpally involved primary teeth should ideally be managed before chemotherapy. Due to a high risk of infection and failure during immunosuppression, extractions are typically preferred for deep carious lesions.

Controversy: Should Primary Teeth Be Saved or Extracted?

Primary teeth with deep caries or pulp involvement present a dilemma during leukemia treatment. Some experts recommend saving primary teeth through pulpotomy/pulpectomy if they are asymptomatic and critical for space maintenance or function, provided the child is not yet immunocompromised. Others argue for extraction as a safer option, given the heightened risk of infection, delayed healing, and failure during chemotherapy-induced immunosuppression [11]. Current consensus leans toward the extraction of non-restorable and pulpally involved primary teeth prior to initiating therapy.

Endodontic Therapy in Permanent Teeth

If required, root canal treatment (RCT) should be performed in a single visit and monitored for symptom resolution at least one week prior to chemotherapy. In cases where symptoms persist, defer definitive treatment until hematologic recovery. Alternatives like pulpectomy or extraction may be considered based on the individual case [34].

Management of Orthodontic Appliances

All fixed/removable appliances, including space maintainers, must be evaluated for hygiene and safety. Active orthodontic treatment should be avoided during therapy and deferred for a minimum of two years post-remission [35].

Assessment of Periodontal and Mobile Teeth

Teeth with poor prognosis, due to mobility, furcation involvement, or persistent infection, should be extracted. Naturally exfoliating primary teeth may be retained if asymptomatic and well-maintained, but should be closely monitored.

Timing and Technique for Extractions

Extractions should ideally be done 10-14 days before starting chemotherapy. Atraumatic techniques promote better healing and reduce systemic infection risk.

Ongoing Oral Hygiene Guidance

Consistent reinforcement of oral hygiene is crucial to prevent mucositis, opportunistic infections, and systemic complications [36].

Emergency-Only Interventions

Elective dental procedures should be postponed during active treatment phases. In case of dental emergencies, coordination with the oncology team is mandatory for antibiotic prophylaxis, transfusion support, or premedication.

Monitoring Hematologic Parameters for Dental Safety

Proper assessment of hematologic parameters is crucial before performing dental procedures in patients with compromised immunity or platelet function. The following table provides guidance on dental management based on absolute neutrophil count (ANC) and platelet count (Table 4) [29,30].

**Table 4:** Dental management based on hematologic parameters in leukemia patients.

Hematologic parameter	Value	Dental management
Absolute neutrophil count (ANC)	>2000/mm³	Proceed without antibiotics
	1000–2000/mm³	Use clinical judgment; consult an oncologist
	<1000/mm³	Only emergency care with oncologist approval and antibiotic coverage
Platelet count	>60,000/mm³	Standard procedures acceptable
	<60,000/mm³	Avoid invasive treatment; an oncologist consultation is essential

Routine dental check-ups every three months are recommended throughout treatment and survivorship to detect and manage emerging oral health concerns early. A proactive, patient-centered dental protocol is fundamental for supporting both oncologic outcomes and oral quality of life in pediatric leukemia [33].

Current knowledge gaps and future directions in pediatric oncodentistry

Despite growing awareness, several clinical and research gaps continue to hinder the optimal integration of dental care into pediatric oncology. Bridging these gaps requires collaborative research, improved data-sharing mechanisms, and precision-focused protocols that align dental care with systemic cancer treatment (Table 5).

**Table 5:** Current knowledge gaps and future directions in pediatric oncodentistry. AML: acute myeloid leukemia; ALL: acute lymphoid leukemia; CAR T-cells: chimeric antigen receptor T-cells; TKIs: tyrosine kinase inhibitors; BMT: bone marrow transplant; WGS: whole genome sequencing.

Focus area	Current gaps	Future directions	Reference
Research scale and data gathering	The low incidence of childhood cancer restricts the extensive clinical data available for oral health linkages.	Multi-institutional data-sharing projects and big data registries help combine oral health factors and outcomes.	Major et al. (2020) [37]
Supportive care integration	Inconsistent dental care inclusion into overall supportive oncology programs.	Strengthen the part oral health plays in survivorship care, infection prevention, nutritional status, and quality of life outcomes.	Freedman et al. (2023) [38]
Pediatric acute myeloid leukemia management	High-intensity treatments cause underreported and under-monitored oral problems.	Reduce treatment-related oral toxicity using immunotherapies and precision medicine; include dental examination in acute myeloid leukemia clinical studies.	Iyer (2025) [39]
Risk stratification and prevention in acute lymphoid leukemia	Not always investigated in risk-based methods are the oral health effects of chemotherapy and stem cell transplantation.	Investigate oral side effects in new treatments like CAR T-cells and TKIs; explore preventative dental procedures during acute lymphoid leukemia therapy periods.	Elgazar & Constantinou (2024) [40]
Ethics and access in leukemia therapy	Inequalities in oral treatment and rehabilitation following BMT; ethical lapses in dental risk-benefit analyses.	Research oral side effects in novel therapies such as CAR T-cells and TKIs; look at prophylactic dental care during acute lymphoid leukemia treatment cycles.	Panuciak et al. (2022) [41]
Genomic and molecular advancements	Limited integration of genetic profiling in estimating oral disease risk during leukemia therapy.	Use genetic categorization technologies (like WGS and methylation profiling) to customize oral care routines and foresee problems.	Walter et al. (2025) [42]

## Conclusions

With improved survival in pediatric leukemia, managing oral complications has become essential. Issues such as mucositis, infections, and dental anomalies affect nutrition, comfort, and treatment adherence. Early dental intervention and continued care across all treatment phases help reduce these risks.

Importantly, children with leukemia benefit most from integrated, preventive-focused dental care within primary settings, not only in specialized clinics. A multidisciplinary approach, emphasizing preventive oral care and strong collaboration between dentists and oncologists, is key to ensuring better outcomes, timely diagnosis, and continuity of care.
